# CTA and DTI combined with robot-assisted puncture external drainage improves clinical efficacy for mild basal ganglia haematoma

**DOI:** 10.3389/fneur.2025.1617605

**Published:** 2025-12-01

**Authors:** Changpin Liao, Guiying Pan, Lide Huang, Wei Wei, Xianfu Wei, Rusli Bin Nordin, Zhen Lu, Jing Ye, Shengde Nong

**Affiliations:** 1 Department of Neurosurgery, Baise People's Hospital, Baise, China; 2 Faculty of Medicine, MAHSA University, Kuala Langat, Selangor, Malaysia; 3 Department of Oncology Radiotherapy, Affiliated Hospital of Youjiang Medical University for Nationalities, Baise, China; 4 Key Laboratory of Research and Development Clinical Molecular Diagnosis for High-Incidence Diseases of Baise, Baise, China

**Keywords:** robot-assisted, puncture and drainage, medicinal conservative therapy, mild basal ganglia hematoma, clinical efficacy

## Abstract

**Background:**

Robot-assisted puncture and drainage have been increasingly popular as surgical interventions for moderate basal ganglia hematomas; nevertheless, there is scant clinical evidence of their effectiveness in patients with mild basal ganglia hematomas (hematoma volume ≤ 15 mL). This study examined the safety and efficacy of CTA and DTI combined with robot-assisted puncture and drainage for mild basal ganglia hematomas.

**Methods:**

We analyzed the clinical data (general information, short-term efficacy, and long-term efficacy) of 104 patients with mild cerebral hematoma, of whom 62 opted for medicinal conservative treatment (control group) and 42 opted for CTA and DTI combined with robot-assisted puncture drainage (experimental group).

**Results:**

Compared with the control group, patients in the experimental group had a shorter hospital stay, a lower incidence of pulmonary infections, and a significantly lower frequency of antibiotics and mannitol within 3 days after surgery. In addition, patients in the experimental group had a significantly lower amount of residual hematoma within 3 days after surgery, which was completely resolved after 7 days, significantly shorter than the average hematoma subsidence time of 21 days in the control group. The clinical efficacy of the experimental group was better than those of the control group at 30 days, 3 months and 1 year.

**Conclusion:**

The management of mild basal ganglia hematomas by CTA and DTI combined with robot-assisted puncture drainage holds significant potential for clinical implementation.

## 1 Background

Hypertensive intracerebral hematoma (HICH) is the predominant form of hemorrhagic stroke and may result in severe ramifications (1, 2). Prior epidemiological research has demonstrated that basal ganglia hematomas are the predominant form of HICH (3, 4). The hematoma of basal ganglia hematomas exerts pressure on the corticospinal tract (CST) of the posterior limb of the internal capsule, which can lead to deformation, displacement, or rupture of the CST, resulting in severe neurological impairment and significantly increasing the patient’s mortality and disability (5). Some researchers believe that early hematoma removal reduces the potential damage caused by the hematoma and its metabolic by-products, and that the robot-assisted puncture and drainage is one of the main methods for early hematoma removal (6, 7). This procedure has been increasingly used in the treatment of moderate cerebral hematoma by its high precision and low trauma in removing hematomas, which significantly improves the prognosis of patients (8–10).

What about mild basal ganglia hematomas (hematoma volume ≤ 15.0 mL)? Owing to their non-lethal nature, mild basal ganglia hematomas are typically managed conservatively with medication. However, the clinical outcomes are usually poor. With the continuous progress of imaging technology and robotics, the application of robot-assisted puncture external drainage has gradually expanded. Whether this procedure has potential application value in mild cerebral hematoma has become a hotly debated in clinical. Currently, the Clinical evidence on the efficacy of robot-assisted puncture and drainage for mild basal ganglia hematomas remains limited (8). This retrospective study examined the clinical efficacy of CTA and DTI combined with robot-assisted puncture and drainage for mild basal ganglia hematomas, aiming to provide reference data for treatment.

## 2 Methods

### 2.1 Study protocol and patient selection

**Figure 1:**
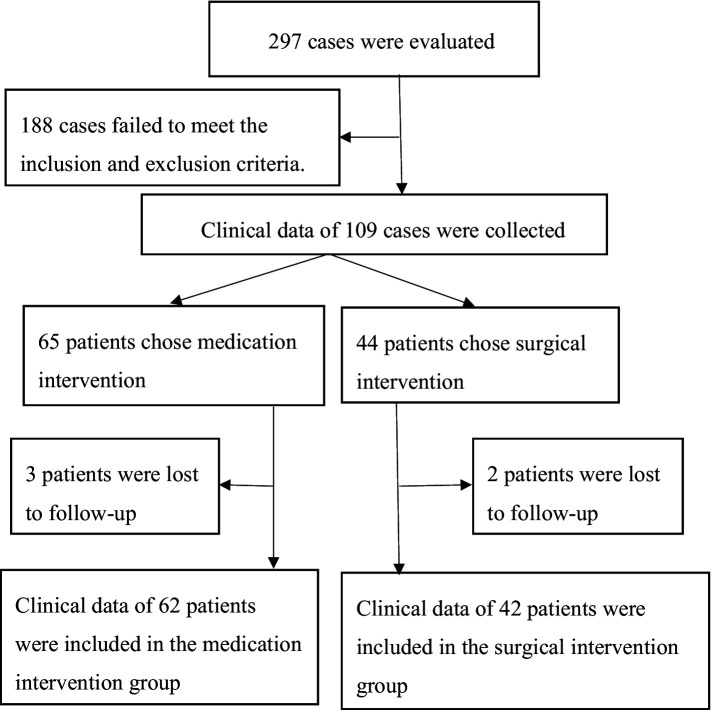
The flow diagram for patient selection.

This retrospective case–control study was approved by the Ethics Committee of Baise People’s Hospital and included patients diagnosed with mild basal ganglia hematoma according to the 2015 “Guidelines for the Management of Spontaneous Intracerebral Hematoma: AHA/ASA Guidelines for Healthcare Professionals.” This retrospective study was conducted between January and December 2021 at the Department of Neurosurgery, Baise People’s Hospital, Baise, China. Patients selected their treatment approach based on their condition, clinical judgment of the physicians, and understanding of the treatment options. Based on the treatment plan, patients were divided into a control group and an experimental group. This study rigorously followed the inclusion and exclusion criteria for participant screening. It utilized consistent standards and methodologies to thoroughly assess and analyze the clinical data of all patients, thereby mitigating the impact of selection bias. The flow diagram for patient selection is as follows (Figure 1).

### 2.2 Inclusion criteria

(1) History of hypertension (SBP ≥ 140 mmHg or DBP ≥ 90 mmHg); (2) Onset within 24 h; (3) Limb muscle strength of ≤grade 3; (4) Basal ganglia haematoma (thematoma volume ≤ 15 mL, measured using the Remebot surgical planning system), with or without involvement of the ventricular system, but without obstructive hydrocephalus, as confirmed by computed tomography (CT) assessment; (5) Diffusion tensor imaging (DTI) confirmed that basal ganglia hematoma compresses the CST’s posterior limb of the internal capsule, leading to deformation, displacement, or rupture, and with limb muscle strength below grade 3.

### 2.3 Exclusion criteria

(1) Patients diagnosed with coagulation problems, brain tumors, cerebrovascular malformations, or cerebral hematomas resulting from aneurysm rupture; (2) Patients deemed unsuitable for surgery due to other physical or mental conditions; (3) Patients with inadequate clinical data.

### 2.4 Clinical data

The collected data encompassed gender, age, Activities of Daily Living (ADL) score, National Institutes of Health Stroke Scale (NIHSS) score, Charlson Comorbidity Index, hematoma volume, muscle strength in both upper and lower extremities at admission, duration of hospital stay, frequency of antibiotic and mannitol administration, incidence of postoperative complications (such as pulmonary), degree of residual hematoma after 3 days, presence of complete hematoma resolution within 7 days, and long-term clinical efficacy at 30 days and 3 months (assessed via ADL score, NIHSS score, and limb muscle strength) along with the Modified Rankin Scale (MRS) score after 1 year. The ADL and MRS scores were assessed by inquiring with the individual or their carer regarding their independence in executing self-care activities, including dressing, grooming, eating, and toileting, and assigning a score depending on their proficiency in these tasks. Muscle strength in the limb is assessed using manual muscle testing, wherein a physician applies resistance to a muscle group and evaluates the strength based on the patient’s capacity to withstand the resistance.

### 2.5 Robot-assisted puncture and drainage utilizing multimodal medical image fusion

All patients in the experimental group underwent CTA and DTI combined with robot-assisted puncture and drainage within 24 h at onset, followed by urokinase administration to facilitate hematoma evacuation.

**Figure 2:**
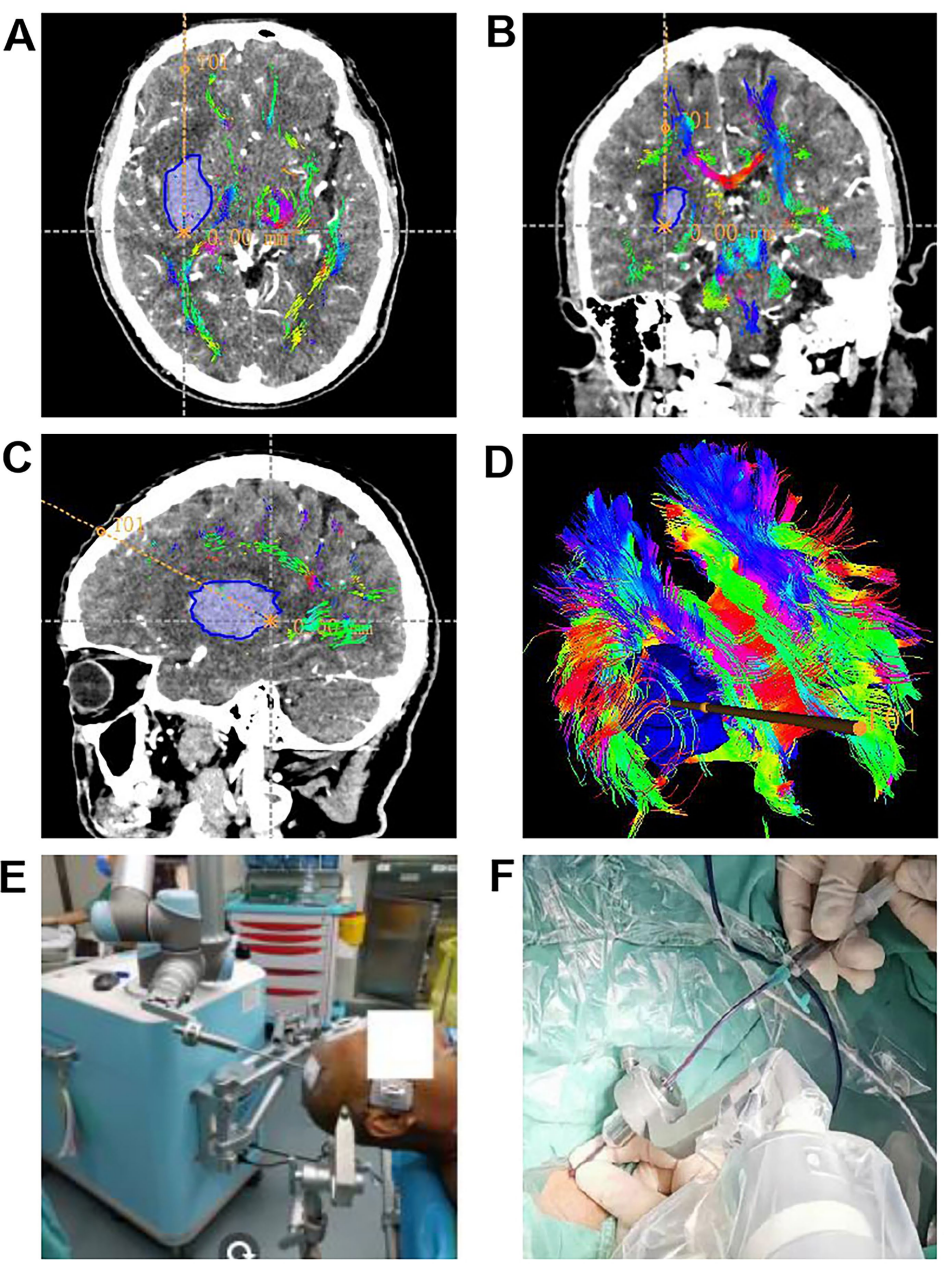
Surgical planning pathway and surgical procedure diagram. **(A–D)** The Remebot surgical robot designs a predefined surgical trajectory based on CTA and DTI data, ensuring avoidance of blood vessels while navigating through DTI-identified gaps to reach the target site. Images are presented in axial **(A)**, coronal **(B)**, sagittal **(C)** views, along with a three-dimensional reconstruction **(D)**. **(E,F)** Surgical intervention treatment procedure.

1. After CTA and DTI scanning of the brain, the resulting image data is transferred in DICOM data to the Remebot surgical planning system. Through this system, the vessels and CST were fused and the surgical trajectory was planned based on the fused data. Specifically, the hematoma center was used as the target point, the maximum hematoma level was selected as the puncture plane, and the surgical trajectory was designed to avoid the CST, vessels, and other critical functional areas (Figures 2A–D).
2. Following successful general anesthesia, a Mayfield head frame secures the patient’s head. The Remebot robot’s camera captures the patient’s head and facial information. Registration and alignment are then performed to ensure alignment accuracy of <1.0 mm. The cranial entry site is confirmed according to the surgical plan. Disinfection and draping procedures are then performed according to standard protocols. A longitudinal incision is approximately 1.0 cm in length on the scalp at the cranial access site. A bone hole with a diameter of approximately 0.5 cm is drilled using a cranial drill, and a 0.5 cm K-wire is inserted to penetrate the dura mater. Under robotic arm guidance, a drainage tube (a soft silicone catheter with a diameter of 0.5 cm) is implanted along the pre-planned surgical pathway. Once the drainage tube reaches the target point, a portion of the hematoma is aspirated under negative pressure using a 5.0 mL syringe (Figures 2E,F).

**Figure 3:**
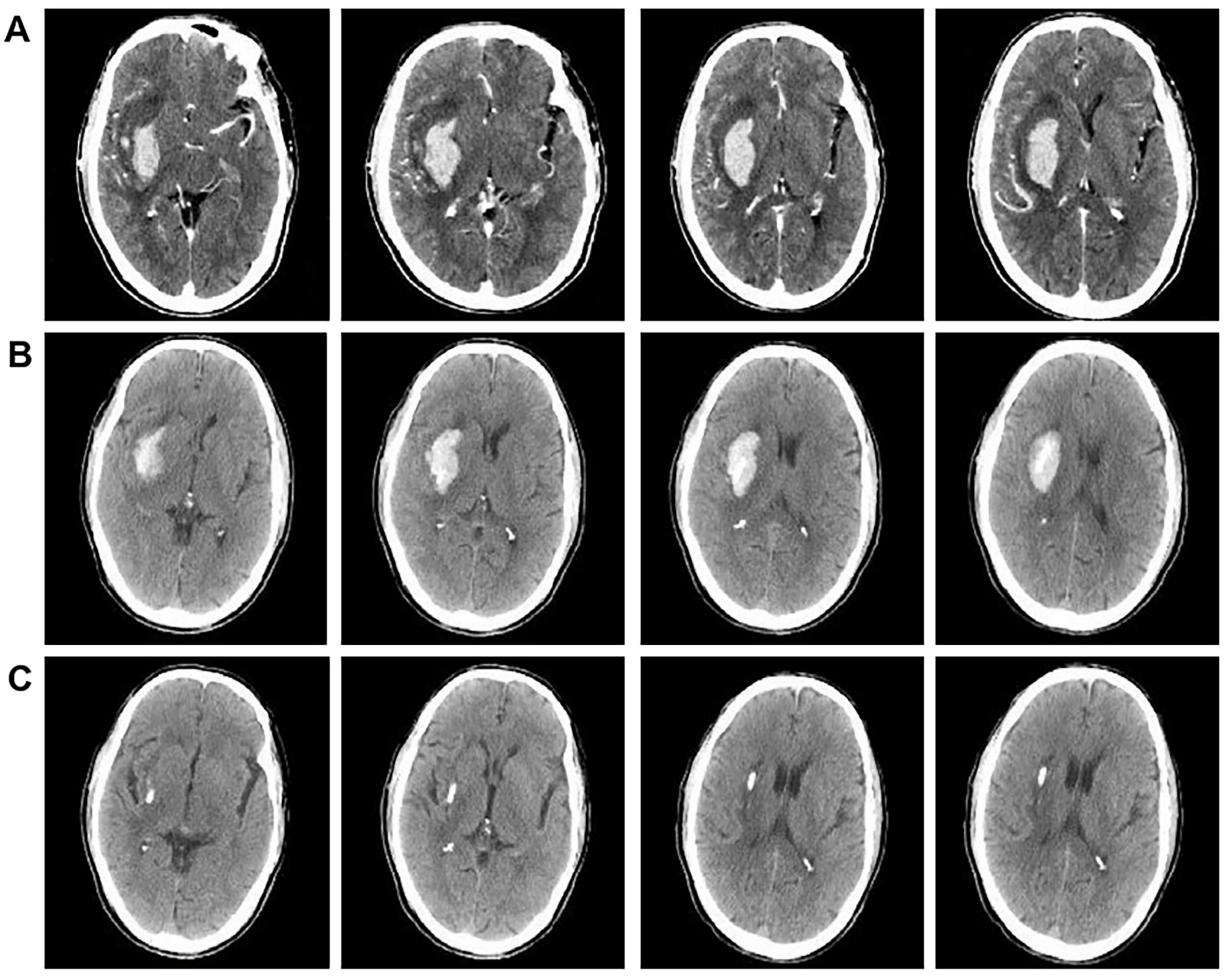
CT comparison images before and after surgical intervention. **(A)** Brain CTA scan images of patients upon admission. **(B)** Brain CT scans of patients 24 h post-surgery. **(C)** Brain CT scans of patients 3 days post-surgery.

When significant resistance is encountered or the volume of hematoma removed reaches 60%, the procedure is concluded by ceasing suction of the hematoma and securing the drainage tube. Subsequently, urokinase thrombolytic therapy is administered. This involves dissolving 20,000 units of urokinase in 2.0 mL of 0.9% saline solution and infusing it into the hematoma cavity via the drainage tube. The drainage tube is then closed, and urokinase is retained for 2–3 h. Afterward, the drainage tube is reopened to drain the urokinase and liquefied hematoma. This procedure is performed 2–3 times daily for 2–5 days. Urokinase thrombolytic therapy was immediately discontinued, and the drainage tube was removed once the cerebral hematoma had completely resolved. The absorption of cerebral haematoma was monitored by the first brain CT scan within 24 h after surgery (Figure 3B) and subsequent CT scans every 2–3 days, depending on the patient’s condition (Figure 3C). Other treatment measures and pharmacological interventions were consistent with the medication intervention group, with specific details as follows.

### 2.6 Medicinal conservative therapy

**Figure 4:**
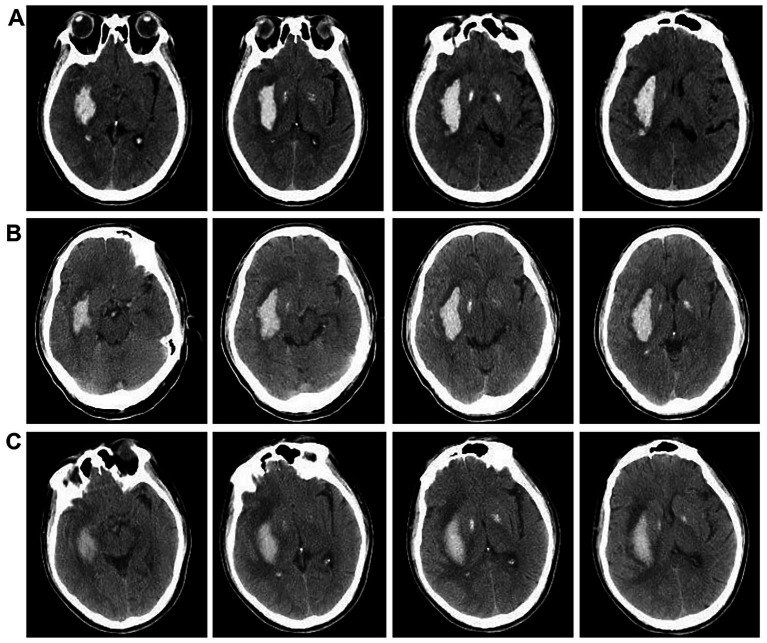
CT Comparison images before and after medication intervention treatment. **(A)** Brain CTA scan images of patients upon admission. **(B)** Brain CT scans of patients after 3 days post-admission. **(C)** Brain CT scans of patients after 7 days post-admission.

Medication intervention encompasses tranquil relaxation, airway clearance, monitoring of body temperature, blood pressure, and respiration rate, as well as managing cerebral edema, among other aspects. Typically, regardless of whether antihypertensive medications are administered intravenously or orally, the average reduction in arterial pressure during the initial phase (≤1 h) remains within 25.0% of the baseline level, achieving a safe threshold (approximately 160/100 mmHg) within 2–6 h, and returning to normal levels within 24–48 h. If a critical organ experiences ischemia during the process of lowering blood pressure, the duration may extend to 1–2 weeks. Furthermore, the active management of brain edema and a decrease in intracranial pressure are critical components of the therapy for acute cerebral hematoma. This trial administered 3–5 mL/kg of mannitol intravenously within 15–30 min to patients exhibiting signs of intracranial hypertension, including headache, vomiting, and papilledema, effectively managing brain edema and lowering intracranial pressure, resulting in favorable clinical efficacy. Furthermore, dietary support, neuronal health, maintenance of fluid and electrolyte equilibrium, and proactive avoidance and management of associated problems are critical elements of the therapeutic approach. Figure 4 shows the therapeutic efficacy of medication intervention.

### 2.7 Statistical analysis

Determination of sample size for two-sample means, employing a two-sided test with *α* = 0.05 and *β* = 0.2, utilizing a 1:1 sample ratio. Preliminary investigations indicated that the ADL scores were μ1 = 46.25 ± 22.59 and μ2 = 63.75 ± 21.21, with *σ* = 9.0. The PASS15.0 software determines that each group required a minimum of 21 participants. All statistical data analyses were conducted using SPSS software (version 20.0). All data underwent normality and homogeneity of variance tests. For normally distributed data, means and standard deviations (𝑥̄ ± S) were reported, and independent samples t-tests were used to assess differences in group means. For count data, chi-square tests were employed to compare proportions across categories. Univariate analysis of variance was employed to adjust for potential confounding factors such as age, gender, and the Charlson Comorbidity Index. Statistical significance was set at *p* < 0.05, with *p* < 0.01 indicating highly significant differences. We emphasize that while statistical significance is a crucial indicator for evaluating results, clinical relevance is equally important. Beyond *p*-values, we also calculated effect sizes for ADL scores, NIHSS scores, and MRS scores to quantify the magnitude of intergroup differences.

## 3 Results

1. No statistically significant variations in the baseline clinical features were detected between the two groups (*p* > 0.05), as shown in Table 1.
2. No statistically significant variations in muscular strength were detected between the two groups (*p* > 0.05), as shown in Table 2.
3. Patients in the surgical intervention group experienced markedly reduced hospital stays, lower incidence of lung infections, and decreased utilization of antibiotics and mannitol compared with the medication intervention group. Three days post-surgery, the residual hematoma volume was markedly reduced in the surgical intervention group compared to that in the medication intervention group. in patients treated with surgical intervention, the drainage tube was retained for 3–5 days, and the hematoma was entirely resolved by 7 days, which was markedly shorter than the average of 21 days for those treated with medication intervention. The two groups exhibited no significant differences in the other problems (*p* > 0.05), as shown in Table 3.
4. The clinical efficacy after 30 days and 3 months demonstrated a significant difference between the two groups (*p* < 0.05), with the surgical intervention group showing superior efficacy compared to the medication intervention group (Table 4).
5. The 30-day muscle strength in both the upper and lower extremities, as well as the MRS score after 1 year, showed significant differences between the two groups (*p* < 0.05), with the surgical intervention group exhibiting superior efficacy compared with the medication intervention group (Table 5).

**Table 1:** Baseline clinical characteristics by treatment group.

Baseline clinical characteristics		Medication intervention group (*n* = 62)	Surgical intervention group (*n* = 42)	*t*/χ^2^ value	*p*-value
Gender (*n*, %)	Male	36 (58.06)	26 (61.90)	0.153	0.695
	Female	26 (41.94)	16 (38.10)		
Age (in years)		61.50 ± 10.46	60.12 ± 8.94	0.699	0.486
ADL score at admission (points)		46.61 ± 23.15	45.71 ± 24.56	0.190	0.850
NIHSS score at admission (values of median, points)		17.00	20.50	1.637	0.105
Volume of haematoma at admission (ml)		9.98 ± 4.37	11.12 ± 3.77	1.378	0.171
Charlson Comorbidity Index (score)		7.18 ± 4.83	6.24 ± 3.66	1.070	0.287

**Table 2:** Muscle strength by treatment group.

Muscle strength		Medication intervention group (*n* = 62)	Surgical intervention group (*n* = 42)	*Z*-value	*p*-value
Muscle strength in upper extremities at admission (cases)	Grade 0	2	1	−0.893	0.372
	Grade 1	32	17		
	Grade 2	16	16		
	Grade 3	12	8		
Muscle strength in lower limb at admission (cases)	Grade 0	0	1	−1.522	0.128
	Grade 1	24	9		
	Grade 2	20	15		
	Grade 3	18	17		

**Table 3:** Comparison of clinical data between the treatment groups.

Clinical data		Medication intervention group (*n* = 62)	Surgical intervention group (*n* = 42)	*t*/*χ*^2^ value	*p*
Length of stay (in days)		23.29 ± 0.50	13.81 ± 5.71	10.732	<0.001
Frequency of use of antibiotics (*n*, %)		25 (40.32)	4 (9.52)	11.811	0.001
Frequency of use of mannitol (*n*, %)		26 (41.94)	9 (21.43)	4.716	0.030
Residual amount of hematoma within 3 days (ml)		5.68 ± 5.18	2.27 ± 2.69	4.384	<0.001
Residual amount of hematoma in 7 days (ml)		5.03 ± 3.79	0	8.603	<0.001
Related complications (*n*, %)	Pulmonary infection	34 (54.84)	13 (30.95)	5.768	0.027
	Other	3 (4.84)	3 (7.14)	0.245	0.683

**Table 4:** Comparison of 30-day and 3-month clinical efficacy between groups.

Long-term efficacy	Medication intervention group (*n* = 62)	Surgical intervention group (*n* = 42)	*t* value	*p*-value
30-day ADL score (points)	60.24 ± 23.00	80.12 ± 17.02	−5.061	< 0.001
30-day NIHSS score (values of median, points)	13.50	11.00	2.131	0.035
3-month ADL score (points)	63.15 ± 22.49	84.29 ± 10.96	5.648	< 0.001
3-month NIHSS score (values of median, points)	9.50	4.00	3.008	0.003

**Table 5:** Comparison of 30-day clinical efficacy between groups.

Long-term efficacy		Medication intervention group (*n* = 62)	Surgical intervention group (*n* = 42)	*z*-value	*p*-value
Muscle strength in upper extremities at 30-day (cases)	Grade 1	1	0	−2.767	0.006
	Grade 2	20	6		
	Grade 3	21	14		
	Grade 4	19	16		
	Grade 5	1	6		
Muscle strength in lower limb at 30-day (cases)	Grade 1	3	0	−2.420	0.015
	Grade 2	15	3		
	Grade 3	17	10		
	Grade 4	17	22		
	Grade 5	10	7		
MRS score after 1 year	Grade 0	3	6	−2.211	0.027
	Grade 1	7	8		
	Grade 2	10	8		
	Grade 3	20	10		
	Grade 4	10	6		
	Grade 5	12	4		

## 4 Discussion

The CST, which is responsible for controlling voluntary body movements, originates in the motor regions of the cerebral cortex and projects through the posterior limb of the internal capsule to the contralateral spinal cord (11). It plays a crucial role in fine-finger and large-scale limb movements. When a hemorrhagic event occurs in the basal ganglia, such as in the caudate nucleus, putamen, or globus pallidus, the CST in the posterior limb of the internal capsule may be compressed by the hematoma, leading to the displacement, deformation, or disruption of the tract. These injuries impair the normal transmission of motor signals and are a significant cause of contralateral limb hemiplegia in patients with basal ganglia hematoma (5). DTI is a non-invasive technique that reconstructs three-dimensional images of the CST and quantitatively assesses CST integrity *in vivo*. This is achieved by measuring parameters such as the apparent diffusion coefficient, fractional anisotropy (FA), mean diffusivity, and other indicators that reflect the random, non-uniform movement of water molecules in the brain tissue (12, 13). This provides an important reference for developing robot-assisted surgical protocols. Performing robot-assisted puncture and drainage under DTI guidance is a crucial strategy for minimizing further damage to the CST and enhancing limb function in patients (14). Additionally, preventing secondary hematoma enlargement after the procedure is essential for optimizing clinical prognosis. Chan et al. (15) posited that 70% of patients with brain hematomas demonstrate an elevated risk of early rebleeding (within 3 h post-onset), resulting in unfavorable prognoses and elevated fatality rates. Prior research indicates that imaging indicators in CTA scans, including dot-like, black hole-like, and mixed-density signals (resulting from contrast agent extravasation into adjacent tissues or inside the hematoma), can predict hematoma enlargement (16). In addition, the contrast agent in the cerebral vessels presents a high-density image on CT, which can accurately identify the location of the cerebral vessels. Robot-assisted puncture and drainage combined with tissue plasminogen activator (tPA) for intracerebral hematoma evacuation (MISTIE) is widely used in the management of moderate cerebral hematomas. Research indicates that the MISTIE approach is both safe and effective in diminishing hematoma volume, enhancing patient clinical efficacy, and decreasing associated problems (17–19). The MISTIE I-III trials sought to diminish absolute hematoma volume, enhance hematoma clearance percentage, and limit hematoma size to ≤ 15.0 mL. This study focused on patients with small basal ganglia hematomas (≤ 15.0 mL). In this study, we innovatively employed high-sensitivity CTA scans to assess the degree of vascular aggregation, combined with DTI to evaluate CST integrity. Using the Remebot software system, we designed surgical trajectories that bypassed both blood vessels and the CST, effectively minimizing the risk of intraoperative vascular and CST damage. This approach significantly reduces the likelihood of postoperative bleeding and further CST injury.

This study employed vascular imaging points on CTA images to visualize cortical blood vessels, coupled with DTI to reconstruct three-dimensional CST images. A solitary 12F flexible silicone tube was precisely introduced into the hematoma center via the predetermined surgical route in 42 patients with mild basal ganglia hematoma in the surgical intervention group. Ziwei Chen et al. (20) found that in robot-assisted puncture and drainage, using a drainage tube aligned with the long axis of the hematoma for small to medium-sized basal ganglia hematomas results in a postoperative rebleeding rate of 4.76%, and that low-dose urokinase (20,000 units) is both safe and effective for treating cerebral hematoma. To expedite hematoma clearance, 42 patients in the surgical intervention group underwent the same postoperative intracavitary thrombolysis procedure, receiving low-dose urokinase (20,000 units) through a silicone tube. No instances of secondary hematoma enlargement were observed, demonstrating the safety and efficacy of this approach. The residual hematoma volume in the surgical intervention group (2.27 ± 2.69 mL) was significantly smaller within 3 days than that in the medication intervention group (5.68 ± 5.18 mL). Patients in the surgical intervention group achieved complete resolution of the basal ganglia hematoma, with no surrounding edema, within 7 days. In contrast, patients in the medication intervention group had larger residual hematomas after 7 days (5.03 ± 3.82 mL) and exhibited considerable edema in the surrounding cerebral tissues, resulting in symptoms of increased intracranial pressure, such as headache and vomiting. Although patients who received surgical intervention treatment had at least 60% of the hematoma cleared within 24 h post-surgery, 9 cases (21.43%) developed symptoms of intracranial hypertension. These patients require an intravenous infusion of mannitol within 15–30 min to promote dehydration and lower intracranial pressure. The incidence of mannitol administration in patients with surgical intervention was significantly lower than that in the medication intervention cohort, which had 26 (41.94%) instances. A statistically significant difference in the frequency of mannitol use was observed between the two groups (*p* < 0.05). The hematoma in the surgical intervention group was successfully resolved within approximately 7 days, alleviating compression on the CST and promoting expedited recovery of limb motor function in the patients. Despite surgical intervention operations being conducted under anesthesia, which somewhat elevated the risk of pulmonary infection, the total duration of bed rest was 3–5 days, much less than the 7–10 days noted in the medication intervention group. A shorter duration of bed rest enables patients to engage in rehabilitation promptly, which is crucial in reducing the incidence of pneumonia and shortening hospital stays. In the present study, 13 patients (30.95%) in the surgical intervention group developed lung infections, of which 9 patients (21.43%) received antibiotic treatment. In contrast, 34 patients (54.84%) in the medication intervention group developed lung infections, and 26 patients (41.94%) received antibiotic treatment. A statistically significant difference was observed between the two groups (*p* < 0.05). Furthermore, the duration of hospitalization was significantly shorter in the surgical intervention group (13.81 ± 5.71 days) than in the medication intervention group (23.29 ± 0.50 days).

In this study, we also used the DTI technique to reconstruct the 3D image of the CST, and carefully delivered the drainage tube through the CST gap to the haematoma cavity according to a predetermined surgical trajectory with the assistance of the Remebot robot. This approach avoided further CST damage while removing the haematoma. In our investigation, no patient in the surgical intervention group developed new neurological functional impairment events postoperatively. Robot-assisted puncture and drainage is an invasive brain procedure that carries a potential risk of intracranial infections. However, in this study, we strictly adhered to aseptic techniques during surgery, performed regular dressing changes postoperatively, and provided high-quality care, effectively preventing the occurrence of intracranial infections. No intracranial infections occurred in the surgical intervention group. The disparity in the Charlson Comorbidity Index between the two patient cohorts was not statistically significant (*p* > 0.05), indicating that the prevalence of chronic diseases and other health issues in both groups was comparable. Consequently, the Charlson Comorbidity Index did not influence the incidence of associated problems (such as lung infections) in either group of patients with cerebral hematoma. The results indicated that patients in the surgical intervention group demonstrated significantly better short-term outcomes (including time to hematoma resolution and associated complications) and long-term outcomes (such as limb muscle strength, ADL, NIHSS, and MRS scores) than those undergoing conservative treatment.

### 4.1 Limitations of the study

This study was a single-center, retrospective analysis subject to selection bias, which potentially affected the outcomes. The authors meticulously examined all patients with mild basal ganglia hematomas admitted to the Department of Neurosurgery at Baise People’s Hospital from January 2021 to December 2021, adhering strictly to the established inclusion and exclusion criteria. The two patient groups were matched as closely as feasible for gender, age, and clinical indicators, including ADL and NIHSS scores, to reduce the influence of baseline characteristic variability on the study outcomes. This study may not entirely eradicate selection bias; nevertheless, its impact can be mitigated to some degree by the aforementioned procedures, enhancing the credibility and applicability of the study conclusions. Second, the sample size was limited, necessitating multicenter, prospective, randomized controlled trials with larger cohorts. The results of this investigation may serve as reference evidence for larger multicenter randomized controlled trials investigating the treatment of mild basal ganglia hematoma in the future.

## 5 Conclusion

This study indicates that surgical intervention therapy for mild basal ganglia hematomas has promising clinical application potential. Despite several limitations and challenges, the surgical intervention has substantial potential for enhancing clinical efficacy in patients. Future multicenter, prospective clinical studies with diverse datasets will further confirm the long-term efficacy and safety of surgical intervention, thereby facilitating its broader implementation in clinical practice.

## Data Availability

The original contributions presented in the study are included in the article, further inquiries can be directed to the corresponding authors.
